# Long-Term Safety and Efficacy of Single or Repeated Intra-Articular Injection of Allogeneic Neonatal Mesenchymal Stromal Cells for Managing Pain and Lameness in Moderate to Severe Canine Osteoarthritis Without Anti-inflammatory Pharmacological Support: Pilot Clinical Study

**DOI:** 10.3389/fvets.2019.00010

**Published:** 2019-02-05

**Authors:** Quentin Cabon, Marine Febre, Niels Gomez, Thibaut Cachon, Paul Pillard, Claude Carozzo, Nathalie Saulnier, Clément Robert, Véronique Livet, Rodolphe Rakic, Nadia Plantier, Philippe Saas, Stéphane Maddens, Eric Viguier

**Affiliations:** ^1^Université de Lyon, VetAgro Sup, Centre Hospitalier Universitaire Vétérinaire, Marcy l'Etoile, France; ^2^Vetbiobank SAS, Marcy-l'Étoile, France; ^3^Université de Lyon, VetAgro Sup, Interaction Cellule Environnement, ICE, Marcy l'Etoile, France; ^4^INSERM, EFS BFC, UMR1098, Interactions Hôte-Greffon-Tumeur, Ingénierie Cellulaire et Génique, Université Bourgogne Franche-Comté, Besançon, France

**Keywords:** mesenchymal stem/stromal cell, allogeneic, osteoarthritis, long-term follow up, neonatal stem cell, lameness, dogs, NSAID

## Abstract

**Objective:** To explore the long-term safety and efficacy of canine allogeneic mesenchymal stromal cells (MSC) administered intra-articularly as single or repeated injections in appendicular joints of dogs affected by moderate to severe refractory osteoarthritis.

**Study Design:** 22 pet dogs were recruited into a non-randomized, open and monocentric study initially administering one cellular injection. A second injection was offered after 6 months to owners if the first injection did not produce expected results.

**Materials and Methods:** Anti-inflammatory treatment (if prescribed) was discontinued at last one week before the onset of treatment. Each injection consisted of at least 10 million viable neonatal allogeneic mesenchymal stromal cells obtained from fetal adnexa. Medical data was collected from veterinary clinical evaluations of joints up to 6 months post-injection and owner's assessment of their dog's mobility and well-being followed for a further 2 years when possible.

**Results:** Mild, immediate self-limiting inflammatory joint reactions were observed in 5/22 joints after the first injection, and in almost all dogs having a subsequent injection. No other MSC-related adverse medical events were reported, neither during the 6 months follow up visits, nor during the long-term (2-years) safety follow up. Veterinary clinical evaluation showed a significant and durable clinical improvement (up to 6 months) following MSC administration. Eight dogs (11 joints) were re-injected 6 months apart, sustaining clinical benefits up to 1 year. Owner's global satisfaction reached 75% at 2 years post-treatment

**Conclusion:** Our data suggest that a single or repeated intra-articular administration of neonatal MSC in dogs with moderate to severe OA is a safe procedure and confer clinical benefits over a 24-month period. When humoral response against MSC is investigated by flow cytometry, a positive mild and transient signal was detected in only one dog from the studied cohort, this dog having had a positive clinical outcome.

## Introduction

Osteoarthritis (OA) is the most common cause of lameness in dogs older than 1 year and accounts for 20% of all canine referrals (1). Prevalence of age-related diseases such as OA have increased in parallel for dogs and their owners as a consequence largely of medical advances. This increase has been exacerbated also by issues of being overweight and a more sedentary life style. Non-steroidal anti-inflammatory drug (NSAID) administration is still the cornerstone of mild to moderate OA pharmacological management. However, their pharmacokinetic properties necessitate daily or at least repeated dosing over a long period, leading to adverse events that compromises their long-term use (2, 3). Constraints put on owners by a daily administration often leads to non-compliance and in turn treatment failure (Zoetis. Rimadyl Chewable Tablets: Compliance Unleashed. 2015-Available from: http://www.zoetisus.com/products/pages/rimadyldvm/docs/compliance.pdf). Therefore, conventionally treated OA progresses systematically to moderate and severe stages that are difficult to control pharmacologically. Alternatives have been tried such as treatment with hyaluronic acid but efficacy is yet to be tested fully and a recent human study showed little improvement over a placebo (4). Similarly, Platelet-rich plasma (PRP) has been indicated for musculoskeletal problems but this also requires further investigation. Expensive surgical management of OA (i.e., arthrodesis, joint resection of the femoral or humeral head, osteotomy for angular correction) carries a significant risk of morbidity while prostheses are not available for all joints.

Cell therapy with Mesenchymal Stem/Stromal Cell (MSC) is a recent therapeutic approach in veterinary medicine (5). Treatment is characterized by a high therapeutic index and a long-term effect, despite the cells relatively short persistence *in vivo*, due to the multimodal mechanisms that counteract the underlying causes of OA (6). Their action involves a transient interaction with the host microenvironment, through secretion of an array of molecules (cytokines, growth factors, miRNA, lipids, micro-vesicles) and direct cell-to-cell interaction leading to prolonged immunomodulatory, trophic, anti-fibrotic, and anti-apoptotic effects (6, 7). MSC would rather promote indirectly an “endogenous tissue healing” rather than direct tissue differentiation as was initially hoped.

In veterinary medicine use of allogeneic MSC is gradually supplanting autologous MSC for treatment of OA, mainly in relationship with its easier way of use and encouraging clinical results. Single intra-articular (IA) injection of allogeneic adult MSC has shown to be a relatively safe procedure with promising results in dogs (8, 9) and horses (10). Injection of allogeneic MSC could be associated with a moderate acute inflammatory joint response greater than those following autologous MSC infusion (11). Recently, we demonstrated the safe clinical use of neonatal allogeneic MSC following knee surgery and compared their efficacy with NSAID (12). Adverse events (AEs) were mainly transient and usually only a self-limiting inflammatory reaction in the days following injection in dogs and horses, although sometimes more pronounced in horses (12, 13).

Most published studies on MSC treatment of OA have used only the normal standard 6 month follow up. This timeframe may not adequately capture any long-term side effects of MSC or their long-term clinical value. Encouraging results in dogs have been reported following single IA injections of allogeneic adipose-derived MSC (AD-MSC) for OA with improved orthopedic examination scores for lameness and range of motion coupled to owner satisfaction. Harman and coll. performed a 60d randomized, placebo-controlled blind study for the treatment of mild OA in pet dogs (8). More recently, Shah and coll. evaluated over 90 days the efficacy of a single injection of AD-MSC in more than 200 dogs (9). Although the data are interesting in demonstrating feasibility and clinical efficacy, long-term assessment is lacking. Considering that OA is a long-term chronic degenerative disease, affecting generally more than one joint, its management by cell therapy may require more than one IA injection, over the dog's lifetime. Meta-analyses of human clinical studies data have confirmed the long-term safety of MSC use in orthopedics, with no significant incidence of AEs (14–16). However, these studies examined only single injections of autologous MSC. Long-term studies with repeated administration of allogeneic MSC are mandatory in animals before transferring them to the wider market. When considering using a second or multiple injections of allogeneic cells, despite the relative immune-evasiveness of MSC (17), it is important to evaluate the risk of eliciting an immunological response against donor cells or any constituent of the product such as fetal calf serum (FCS) (18). Pezzanite et al. detected in recipient sera cytotoxic alloantibodies directed against donor peripheral blood lymphocytes after intradermal administration of a high dose of bone marrow-derived MSC (19), suggesting an alloimmune response against MSC. The same sera displayed cytotoxicity against MHC-I mismatched MSC (20). The same group showed that MHC-I and II-mismatched bone marrow-derived MSC could induce T cell proliferation in a one-way MLR assay. Immune responses highly depend on the cell type, the route of administration, the timing of re-stimulation and should be evaluated specifically for each treatment protocol. Typically, studies have reported a biological humoral immune response using adult-MSC re-injected 1 month after initial stimulation (19). To date no canine studies have been published.

We have conducted a 24-month study to evaluate the long-term safety and efficacy of single and an optional secondary IA injection, separated by 6 months, of allogeneic canine neonatal MSC in pets suffering from moderate to severe OA for which there was no other acceptable therapeutic approach available. **Primary outcomes** were determined by veterinary clinical evaluation of joints up to 6 months post-injections and long-term surveillance for AEs **Secondary outcomes** were evaluated by 1- global owner assessment of their dog's mobility and satisfaction up to 2 years after the final injection 2- owner reports of any medical conditions arising during the 2 years post-treatment period. 3- Evaluation of NSAID independence and recurrence of lameness up to 2 years. We also investigated humoral immune response to neonatal allogeneic MSC administration by measuring the presence of alloantibodies against MSC antigens in recipient dogs. In this NSAID free protocol, we establish the clinical feasibility of repeated IA dosing at 6 months of allogeneic neonatal MSC in dogs with OA. No serious clinical AEs were reported that could be attributed to MSC. Despite the low number of dogs evaluated, we were able to show a significant beneficial effect of MSC in dogs with moderate to severe OA affecting elbows and hips over a 12-month period. A sub-group of dogs received a second injection of MSC, 6 months apart. No humoral immune response against MSC was detected after the first injection, while after a second exposure a mild and transient response was observed in 1/5 re-injected dogs. Since no adverse clinical response was associated with this detection of an immune response, its clinical relevance remains debated.

## Materials and Methods

### Ethical Statement

Dogs enrolled had been referred by their practitioner to the VetAgro Sup Companion Animal Hospital for an orthopedic consultation. This compassionate study was designed based on a critical review by the VetAgro Sup ethical committee (not mandatory under French regulations) and implemented in accordance with University regulations. All dog owners received detailed procedural information. Specific prior written consent from owners including a retraction delay period was obtained.

### Population Study and Criteria of Eligibility

Dogs should present with moderate to severe OA involving at least one joint and one or more reasons why other medical or surgical options have been excluded whilst taking into consideration the owners' wishes. No restrictions were placed on weight, age, sex, or breed. Except that OA, animals must be in good health and free from infectious disease or malignant neoplasia. If such problems were recorded in the dog's medical history, the decision to include the dog was taken collegially on a risk/benefit-based approach. At the time of enrolment, an initial examination of the joint was performed by a specialized surgeon. For dogs with bilateral OA, each joint was evaluated individually. Clinical scoring integrated four parameters evaluated with equal weight: lameness on walking, pain upon joint palpation, pain upon joint manipulation, and local temperature compared to a contralateral limb. Each variable was graded from 1 to 4, leading to a clinical score from 4 to 16 with the highest score reflecting the worst clinical condition. Practitioners were allowed to use intermediate scoring for each parameter when appropriate. A global score equalling 4 corresponds to a healthy joint; from 4.5 to 8 to mild OA, from 8.5 to 12 to moderate OA and from 12.5 to 16 to severe OA (Supplementary Table 1). Front and profile X-rays were taken to confirm OA diagnosis. Both clinical and radiographic findings were integrated to grade OA severity (Table 1), as recommended by the recent recommended OA clinical scoring system (21).

**Table 1:** Grading of radiographic findings based on blinded evaluation of x-rays.

**Radiographic score**		**Radiographic findings**
0	HEALTHY	Normal joint
1	DIM	Radiographic evidence of instability; no degenerative change (no osteophytes)
2	MILD	Mild degenerative change (occasional osteophytes)
3	MODERATE	Moderate degenerative change (osteophytes, subchondral sclerosis)
4	SEVERE	Severe degenerative change (osteophytes, subchondral sclerosis, bone remodeling)

Previous surgery of the affected joint was not an exclusion criterion if surgery was performed at least 1 month before inclusion and if the animal still showed persistent lameness. Therapies such as NSAID and short action steroids had to be discontinued at least 1 week and long-acting steroids for at least 6 months prior to entry (22).

### Study Protocol

**Figure 1:**
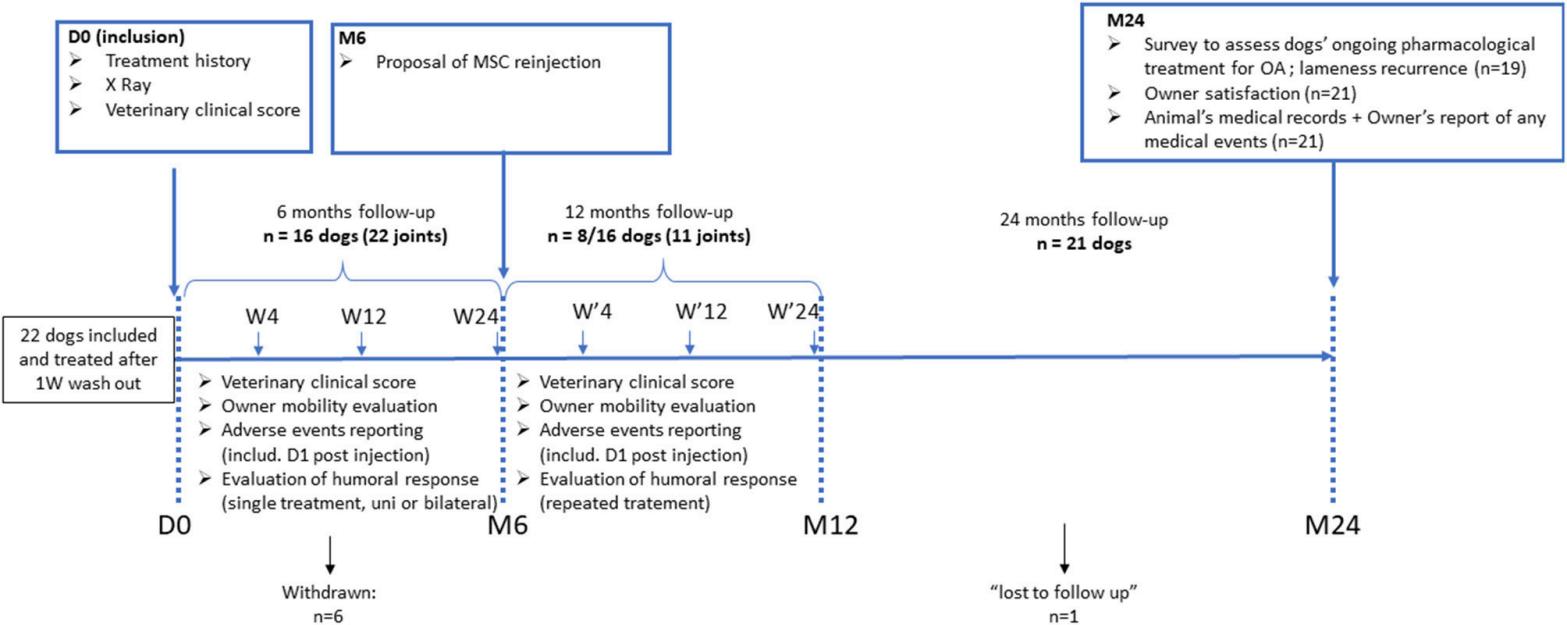
Schematic overview of the study design.

This study was designed as an open-labeled un-controlled monocentric study, using pet dogs diagnosed with moderate to severe OA and having persistent non-treatable lameness for more than 3 months. Details of the study are recapitulated in Figure 1. All dogs received a single intra-articular injection of neonatal canine allogeneic MSC in a maximum of 2 OA joints. There were 3 follow up examinations at the investigational site, programmed at week 4 (W4), W12, and W24 post-injection to evaluate clinical safety and efficacy. Blood samples were collected at each time point to detect immune responses against the cellular product. Six months after, owners were offered a second injection if they agreed to adhere to the same follow up protocol. Two years post-injection, the dog's medical records were reviewed, and owners were asked by email or phone to evaluate their perceived efficacy and treatment safety.

### Cell Product Preparation and Qualification

MSC were isolated from neonatal tissues recovered from medically necessary Cesarean-sections (C-Section), at full-term pregnancy, performed by surgeons from the Reproduction Department of VetAgro Sup (Marcy l'Etoile, France). Extensive adventitious agent screening (including virus, parasites and mycoplasmas) was realized on the bitches' blood sample and on neonatal tissue biopsies collected during dissection. Batches of MSC used in this study were manufactured and characterized as previously described (12, 23). Briefly, cells displayed a conventional phenotype for canine MSC (i.e., CD44+; CD29+; CD90+; MHC2–; CD45–; CD34–), differentiated into 3 mesodermal lineages *in vitro* (adipogenic, osteogenic, and chondrogenic). Evaluation of indoleamine 2,3-dioxygenase (IDO) expression (Ehrlich's assay) upon exposure to the pro-inflammatory cytokine interferon- gamma (IFN-γ) was used as the cellular release criteria as recommended by the guidelines of International Society for Cellular Therapy (24). The expression of MHC-I (clone H58A, Monoclonal Antibody Center) and MHC-II was evaluated before and after *ex vivo* stimulation of MSC by recombinant IFN-γ (canine IFN-γ; R&D System; 5 ng/ml for 3 days) by flow cytometry analysis. Sterility tests were also performed by an independent accredited laboratory on cellular batches before storage in liquid nitrogen. The maximum passage number of the cell batches used in this study was P4.

Upon the day of injection, MSC products were thawed at 37°C, washed with Dulbecco's Phosphate Buffered Saline (D-PBS, Pan Biotech) and viable cells counted. 10 × 10^6^ viable MSC were re-suspended in 0.5 mL of D-PBS and transferred to a sterile 1 mL syringe. Minimal post-thaw viability release criteria was >80% (range 82–98%). The loaded syringe was sent in an appropriately sterile container to the hospital for injection and used the same day.

### Treatment Protocol

After examination, dogs were sedated (Medetomidine 0.005 mg/kg IV and Propofol 4 mg/kg IV, Morphine or Methadone 0.2 mg/kg IV) and received an intra-articular injection of at least 10 × 10^6^ viable neonatal MSC after surgical preparation of the joint area. Dogs were discharged from the clinic at day 1 (D1), following clinical evaluation. Recommendations were provided to the owners to restrict exercise for 3 days following injection. Medical analgesia (tramadol 5 mg/kg PO) was allowed for 1-week post-injection for dogs showing signs of pain upon discharge. The use of any anti-inflammatory medication was prohibited during the entire course of the 6-month study except in exceptional circumstances. If such prescriptions were given during the 6 months following first or second MSC administration, the dog was excluded from the efficacy assessment but not the safety aspects of the study.

### Safety and Efficacy Outcomes

#### Safety Outcome

Clinical safety was evaluated in three phases 1—**short term assessment** was done by a specialized surgeon at D1 after MSC injection, monitoring any signs of inflammation (heat, pain) 2—**mid-term assessment** was made by both a veterinarian and owners reporting at each follow up time point, noting any pathological conditions occurring during the 6-month period after each MSC injection and 3—**long-term assessment**, at 2 years post-treatment, with a retrospective review of the animal's medical records (from the hospital and veterinary clinics) together with the owners' reports of any AEs.

#### Efficacy Outcome

Clinical efficacy was evaluated in the same manner by vets during follow ups as the one used in the Orthopedics Department of VetAgro Sup upon inclusion. Whenever possible, the vet evaluating the dog was not the one who injected the dog. At the same time points, owners assessed their dog's global mobility before and after treatment based on a 4-stage scale by answering a single question: “Do you estimate that mobility is (1) significantly better- (2) slightly better- (3) not changed- (4) worse.” If dogs were re-injected at W24, they followed the same protocol for the additional months. Two years post-injection, dog's owners were asked to complete a survey to report any other OA-related treatments given plus the recurrence, degree, and persistence of lameness (Survey detailed in Supplementary Data 1). Global satisfaction about the treatment was monitored by 5 possible answers: (1) Very unsatisfied- (2) unsatisfied- (3) no opinion (4) satisfied or (5) Very satisfied.

As veterinary evaluation and owner's assessment were performed independently at the same time points, we examined appraisal coherence. To that end, the level of clinical improvement vs. inclusion was graded for each joint as 1-worse (-1), no difference (0), or improvement (+1) by the veterinarian, based on the global review of the scoring throughout the follow up. This scoring was compared to the owner's evaluation based on the same scaling system. Evaluations were considered as coherent when Abs Δ _(practitionerscorevs.ownerscore)_ = 0; divergent when Abs Δ _(practitionerscorevs.ownerscore)_ = 2.

### Flow Cytometric Crossmatch Analysis of Humoral Response Against Cellular Product

We adapted and validated a cross-match procedure to detect the presence of alloantibodies against MSC in recipient dogs after a single or repeated injection of cells (25). Serum was collected prior to injection of MSC and at the different follow up, frozen at −80°C until further analysis.

First, a positive control serum was developed to validate the analytical approach. Briefly, 3 × 10^8^ MSC, originating from selected MSC's batches used in the study, were stimulated *in vitro* with canine IFN-γ (5 ng/ml for 72 h; R&D System) to increase their immunogenicity (Supplementary Figure 1). Following the treatment, cells were detached, washed three times with D-PBS (250G/10 min). Cells were lysed by 3 freeze/thaw cycles and cell lysate was used in an immunization protocol to produce a polyclonal anti-dog MSC rabbit serum (Biotem, France). Serum titer was evaluated by ELISA using the MSC cell lysate. The positive control consisted in incubating MSC with rabbit polyclonal serum (dilution 1:5), followed by a second incubation with a secondary anti-rabbit IgG-FITC (dilution 1:200) (Southern Biotech). Analysis was performed by flow cytometry. Median fluorescence intensity (MFI) of control rabbit serum (pre-immune serum) was used to determine the background signal. The variance between serum collected from immunized rabbit and the background signal was expressed as a difference [Δ = MFI _(afterimmunization)_-MFI _(pre−immune)_]. A signal was considered as positive if Δ exceeds the background signal + 3 SD (standard deviations) (26).

MSC cultured in basal condition or pre-stimulated with IFN-γ were washed twice in D-PBS and centrifuged (300G, 5 min). MSC were incubated in blocking solution (2% normal goat serum; Sigma Aldrich) in D-PBS for 30 min at room temperature (RT). Cells were centrifuged and washed twice in D-PBS (300G, 5 min). Dog serum (MSC recipient serum collected at the different time points) was diluted 1:5 in D-PBS, and 200 μl was added on the pellet. Cells were re-suspended in the diluted serum and incubated at RT for 30 min. Following incubation, cells were washed twice with D-PBS (500G, 5 min), and 100 μl of secondary antibody (Goat anti-dog IgG-FITC diluted 1:200, Southern Biotech) was added to the cell pellet, mixed, and incubated for 30 min at 4°C. Cells were washed twice in D-PBS (800G, 5 min) and re-suspended in 100 μl of FACS buffer before acquisition by a flow cytometer (Accuri C6, BD PharMingen).

For each sample, fluorescence signals from 20.000 cells were acquired. To account for the variability of the samples, MFI of canine sera (from untreated animals) to donor MSC (resting MSC or IFNγ-primed MSC) were used to determine the background signal for each experimental condition independently. A cut-off value of positivity was set-up for fluorescence signals exceeded the defined background threshold +3SD. The median channel shift of the MFI of the sera collected at each time point (W4, 12, 24) and the MFI of the paired control serum (D0) was expressed as a difference [Δ = MFI _(specifictimepoint)−_MFI _(controlserum;D0)_]. A serum sample was considered “positive” for anti-MSC antibodies if Δ exceeds the cut off.

### Statistical Analysis

Statistical analysis employed the GraphPad Prism 6.0 program (GraphPad Software, USA). Changes in the clinical score over time were calculated using the non-parametric Friedman repeated measures ANOVA on a rank sum test. *Post-hoc* comparisons were made using a Dunn's test. Differences were considered significant for *P* ≤ 0.05.

Comparison of the clinical score evolution between more lame limbs group and less lame limbs group was conducted using two-way repeated measures ANOVA. A *post-hoc* comparison was obtained using Sidak's test.

Baseline comparisons between hip and elbow on clinical scores, weight, age, were produced using the Mann-Whitney *U*-test. Categorical data (gender) were compared using Fisher's exact test.

## Results

### Clinical Information on the Study Cohort

Twenty-two pet dogs were enrolled over a 15-month period and were analyzed in a 2-year safety study with a 95% successful follow up rate. Criteria for study inclusion were: A- intolerance to NSAID (*n* = 4), B-partial or total lack of response to at least one NSAID (*n* = 6) C-medical condition contraindicating long-term prescription of NSAID (*n* = 0) D-owner refusal of surgical options (*n* = 2) E-Owner refusal of NSAID or any other treatment (*n* = 4). Detailed dog information is shown in (Table 2). During the first 6-month period, 6 (27%) dogs (#17–22) were eliminated from the study. Dog#19 included for right hip was withdrawn after lameness and swelling of the right tarsus between W4 and W12. Arthroscopy revealed Osteochondritis Dissecans (OCD) requiring bone fragment removal and a NSAID prescription. Dog#22 was withdrawn after a rupture of the cranial cruciate ligament of the contralateral hind knee between W12 and W24 requiring a NSAID prescription. Dogs#17; 18; and 21 (13.6%) were withdrawn because the owners didn't attend the follow up visit at W24. However, information provided by the owners when contacted did not mention any medical reason related to a lack of efficacy or side effects. Dog#20 received MSC 3 months after TTA surgery on the same knee. He developed an infection on the TTA plate 4 months after TTA surgery requiring the removal of the plate and treatment to enable bone healing. In these cases, a review by the study's medical steering committee didn't find a link to MSC injection. 8 dogs (#4–8;10;12;14) were re-injected at 6 months and none had to be withdrawn during the subsequent 6-month follow up period.

**Table 2:** Description of the dog population.

**Id**	**Age (years)**	**Breed**	**Weight (kg)**	**Gender**	**Affected joint(s)**	**Unilateral (U)/bilateral (B)**	**Inclusion clinical score (/16)**	**Inclusion radiographic score (/4)**	**Mild/Moderate/ severe**	**Agreed to reinject 6 months apart**	**Reasons for inclusion**
**(A) DOGS WHO COMPLETED THE 6 MONTHS STUDY**											
Dog 1	8	Boxer	28.5	m	Hip	B	9/5	4/4	Severe/severe	No	B
Dog 2	5	Boxer	30.1	m	Hip	U	14	3	Severe	No	D
Dog 3	6	Boxer	38.6	m	Knee	U	7	4	Severe	No	E
Dog 4	10	Hungarian	20.4	f	Elbow	B	12/12	4/4	Severe/severe	Yes	A
Dog 5	8	English cocker spaniel	13.5	m	elbow	U	10	4	Severe	Yes	B
Dog 6	10	Labrador retriever	31.2	f	Hip	U	9	3	Moderate	Yes	D
Dog 7	7	Labrador retriever	28	m	Hip	B	9/9	3/3	Moderate/moderate	Yes	E
Dog 8	8	Colley	21.3	f	Hip/Carpal joint	B	8/9	4/2	Severe/moderate	Yes	B
Dog 9	1.5	Boxer	35	m	Hip	U	8.5	1	Moderate	No	A
Dog 10	1	Golden retriever	36	m	Elbow	B	9/9	4/4	Severe/severe	yes	E
Dog 11	2	Labrador retriever	19	m	Hip	U	8.5	1	Moderate	No	B
Dog 12	4	German shepherd	25	f	Hip	U	6	3	Moderate	Yes	A
Dog 13	1	Labrador retriever	29.4	m	Tarsal joint	U	10	2	Moderate	No	B
Dog 14	4.5	Anatolian shepherd	48.5	m	Hip	U	12	4	Severe	Yes	A
Dog 15	1	Bernese mountain	41.7	f	Elbow	B	6/4	3/3	Moderate/moderate	No	B
Dog 16	8.5	Golden retriever	31.6	m	Elbow	U	6.5	3	Moderate	No	E
**(B) WITHDRAWN**											
Dog 17	8	Shar-Pei	20	m	Elbow	U					
Dog 18	*7*	*German shepherd*	*35.2*	*m*	*Knee*	U					
Dog 19	2	cane corso	32.4	f	Hip	U					
Dog 20	*1*	*Bernese mountain*	*46*	*m*	*Knee*	U					
Dog 21	1	German shepherd	35	m	Hip	U					
Dog 22	*5*	*Golden retriever*	*21.4*	*f*	*Hip*	U					

*(A) 16 dogs completed the study. (B) 6 Withdrawn dogs. Gender: m, male, f, female; Reasons for Inclusion A—intolerance to NSAID, B—partial or total lack of response to at least one NSAID specialty, C—medical condition contraindicating long-term prescription of NSAID, D—owner refusal for a surgical option, E—owner refusal of NSAID or any other treatment*.

Sixteen **dogs** (73%) completed the first 6-month period follow up and analyzed in the efficacy study. Breeds included Boxer, German shepherd, Labrador, Golden retriever, Bernese Mountain, Brac Hungarian, English Cocker Spaniel and Anatolian shepherd. Eleven were males and 5 females. Average age of the population was 5.3 years (range 1–10) and average body weight was 29.9 kg (range 13.5–48.5 Kg). Eight out of 16 dogs were receiving NSAID treatment at the time of enrolment (4 elbows, 2 hips, 1 knee, 1 tarsus) but discontinued treatment. Median clinical score at inclusion was 9 (range 4–14) and the median radiographic score was 3 (range 1–4). Ten dogs presented unilateral OA and 6 had bilateral OA. All dogs with bilateral OA received injections in both joints during the same operation under general anesthesia, except dog#1 whose owner asked that the most painful hip was treated first and the second after 6 months.

From the 16 dogs, 22 **joints** were examined: hips (*n* = 11); elbow (*n* = 8); carpal (*n* = 1); tarsus (*n* = 1) and knee (*n* = 1). All joints were defined with moderate (*n* = 11) or severe (*n* = 11) OA based on radiographic findings and clinical examination (Table 2).

At the end of the first 6 months of follow up, 8/16 owners (dogs #4, 5, 6, 7, 8, 10, 12, 14) agreed with a second MSC injection and adhere to the same follow up requirements. Three dogs with bilateral OA (#4, 5, 10) were re-injected in both affected joints, while one dog (#8) was re-injected only in the hip. Four animals given an initial single injection (unilateral OA #5, 12, 6, 14) were re-injected making a total of 11 joints (6 hips, 5 elbows). In all cases, the second injection was realized with another MSC batch (Supplementary Table 2).

### Safety

Safety assessment included all dogs enrolled in the trial study, including withdrawn animals in order not to underestimate any side effects related to MSC injection. Dogs were no longer under anti-inflammatory medication before treatment or underwent a drug washout period.

#### Short-Term Assessment

At (D1) after the first administration, 5/22 (23%) joints presented a worse clinical score increasing from 7.9 at D0 to 10.7 at D1. Clinical changes included both increased local temperatures and signs of pain during joint manipulation. Three hips (#7; 8; 12) were involved, 2 elbows (#16; 17) and 1 carpal joint (#8). Two of those dogs were treated for bilateral OA, one (#8) of which had side effects on both hip and carpal joints, the other dog had only involvement of one hip (#7). For all dogs, symptoms disappeared spontaneously within 48 h with no recurrence. Only one dog#12 was given tramadol upon discharge. Short term safety was also examined after repeated injection. Eight dogs received a second injection 6 months later. Among the 11 joints re-injected (6 hips and 5 elbows), 5 joints did not react. 6 joints, principally hips (5 hips #7;8;12;14;6 and 1 elbow #5) produced a worsened global clinical score. Median scores increased by 28% from 7.2 at reinjection to 10.7 the day after. Three (#7;8;12) of those 6 joints, all hips, had also presented a reaction after first injection. The elbow of dog #5 hadn't reacted for the first injection. ***Mid-term*** ***assessment***- Over the 6-month period following the initial injection, or repeats, no serious adverse events were reported either by the owners or by the vets. Long-Term Assessment

AEs were monitored up to 2 years after initial injection. Nine dogs did not present again at investigation site. Clinical records collected from their vets did not report any AEs except for 3 dogs (#12;13; 17) for which data were lacking since they were lost to follow up. Seven dogs returned to investigational site between the last follow up and 2 years for medical reasons unrelated to OA. Four dogs then received repeated injections and 3 had a single injection. Their clinical electronic records were analyzed. Dog #4 developed a localized demodicosis infection at M23. Dog #1 (boxer) presented with a suspected testicular tumor at M11 and castrated at M18. Dog#3 developed signs of a mastocytoma at M5. Dog #2 died of heat stroke during an intensive walk at M20. All of the aforementioned setbacks were not considered by the study's steering committee to be related to the treatment. In addition to veterinary records, 21 safety profile surveys (21/22) were completed by owners and returned to the clinical investigational site in ~2 years (range 18 months−3 years). Only one owner refused to answer the questionnaire after his pet's death (Dog#2). Other owners did not report any AEs.

### Efficacy

Dogs who received MSC treatment once or twice were analyzed separately using joint-related parameter (i.e., Clinical scoring), or jointly for individual-related parameters (i.e., NSAID prescription). Dogs excluded during the first 6 months were not analyzed.

### Clinical Evaluation of Joints

#### Single IA Injection/Joint

**Figure 2:**
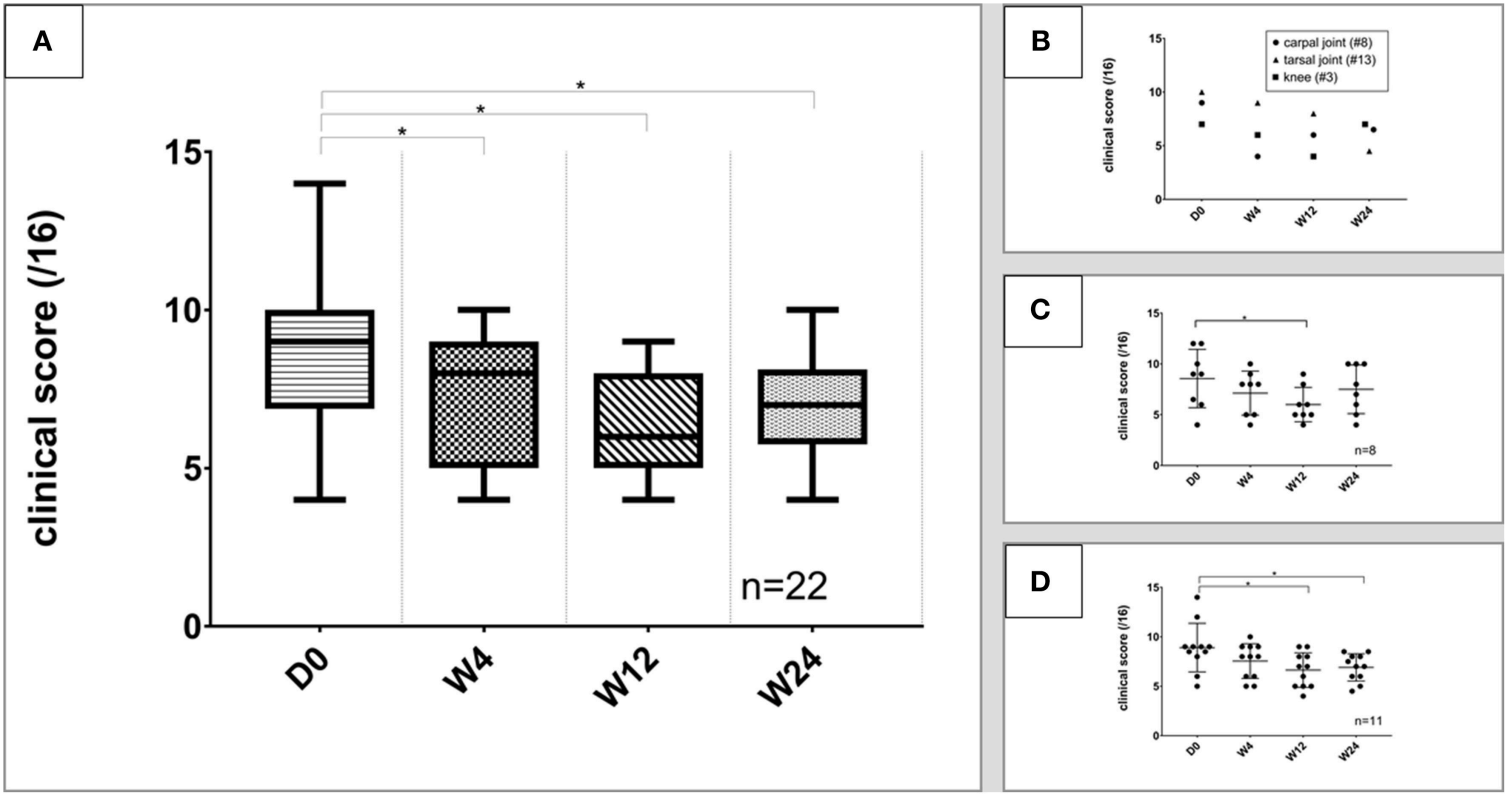
Evolution of clinical scores. ^*^Significant difference (*p* < 0.05) between each timepoint and day 0. Data are presented as median and range. **(A)** Clinical score evolution of all joints; **(B)** clinical score of the unilateral carpal, tarsal and knee joints; **(C)** evolution of clinical scores of elbows; **(D)** evolution of clinical scores of hips. These figures show a improvement of the clinical score throughout the study for all tested joints.

Clinical evaluation of each of the 22 joints was performed at each of the follow up visits (W4, W12, W24) and compared with D0 (inclusion). All joint types were considered, and median clinical scores improved significantly at all-time points (W4: 8 (4–10) *p* = 0.02/W12: 6 (4–9); *p* = 0.0001/W24: 7 (4–10); *p* = 0.0088) vs. D0 (9 (4–14)) (Figure 2A). Clinical evolution score analysis of the unilateral carpal, tarsal, or knee joint (1 animal/joint) is represented separately to facilitate analysis (see Figure 2B). Elbows (*n* = 8) and hips (*n* = 11) are the most representative joint in our study. We aimed at evaluating if a sub-analysis per joint may bring additional information. Results showed a significant improvement of clinical scores at W12 for elbows (*p* = 0.0201) and hips (*p* = 0.0499), while significance was observed only for hips at W24 (*p* = 0.0397) (Figures 2C,D). To rule out possible population heterogeneity between the hip and elbow groups with the observed outcome, the main characteristics of both populations were verified. Age at inclusion (*p* = 0.9211), gender (*p* = 0.8030), weight (*p* = 0.8981), and clinical score at inclusion (*p* = 0.9156) were found not to be statistically different between hip and elbow groups.

**Figure 3:**
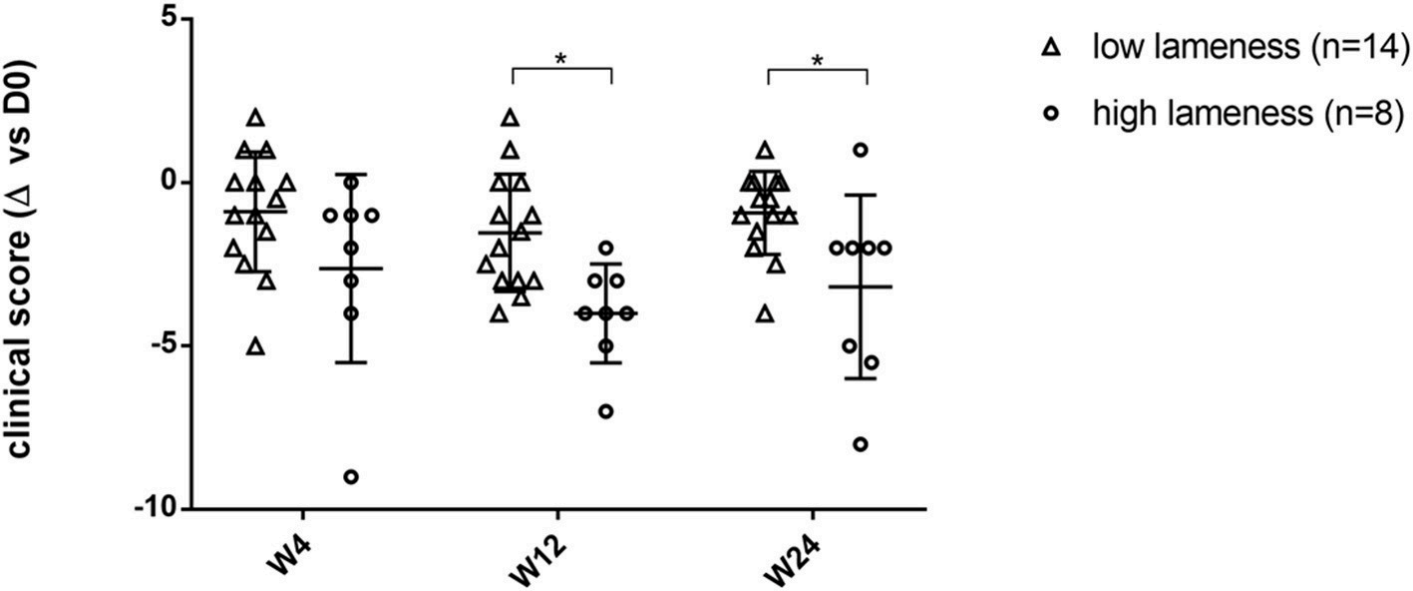
Comparison of the clinical score evolution between more lame limbs group and less lame limbs group after treatment during the 6-month follow-up period. ^*^Significant difference (*p* < 0.05) between low lameness and high lameness groups at each time point. This figure shows that the clinical score evolution is significantly more changed in joints severely affected.

The degree of lameness can affect the ability to observe improvement in clinical scores (1/4 of the clinical score weight) (27, 28). Therefore, we examined results where the joint lameness was considered as “low” (i.e., walk parameter lower than 3; *n* = 14 joints) or “high” (lameness parameter higher or equal to 3; *n* = 8 joints). As expected, data show a significant difference of the clinical improvement of dogs with high lameness at inclusion compared to animals with low lameness (*p* = 0.02 at W12; *p* = 0.0371 at W24) (Figure 3), with animals with high score lameness showing better improvement.

#### Repeated IA Injection/Joint

Of the 16 dogs included in the study, 8 received a second injection of MSC in the same joint(s) (#4–8, 10, 12, 14). A total of 11 joints were re-injected. Interestingly, joints which were re-injected had a significantly higher clinical scores (*p* = 0.0007) at the 6-month follow up point than joints which were not re-injected (*n* = 11), providing a rational for re-injection (data not shown).

**Figure 4:**
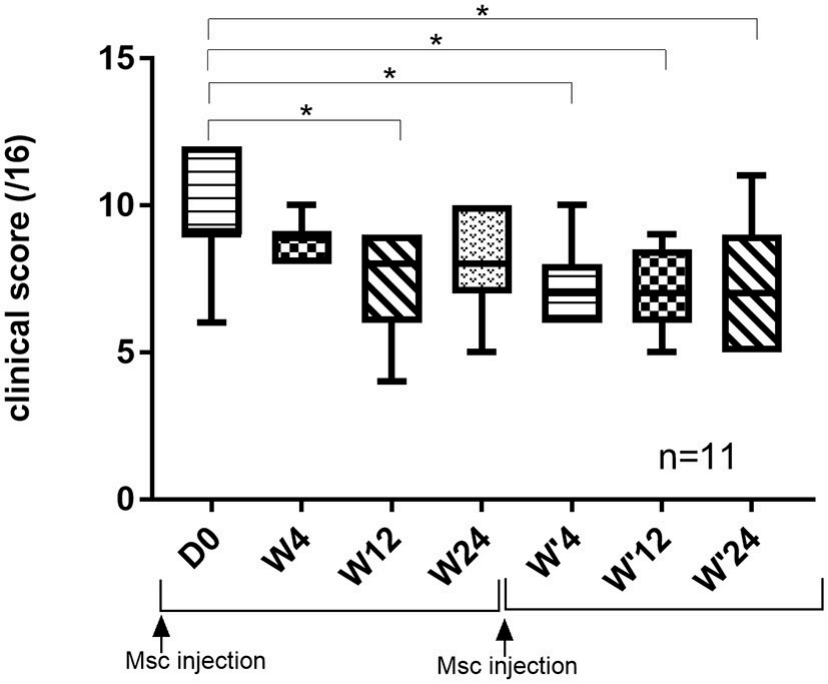
Evolution of clinical score of joints re-injected over 12 months. ^*^Significant difference (*p* < 0.05) between the different time points and day 0.

While clinical scores were not significantly different at W'4, W'12, and W'24 compared to W24 (2d injection), our results showed a significant improvement of the clinical score at W'4 (*p* = 0.0019), W'12 (*p* = 0.015), and W'24 (*p* = 0.016) compared to the inclusion score (D0) (Figure 4).

### Owner Outcome Assessments

**Figure 5:**
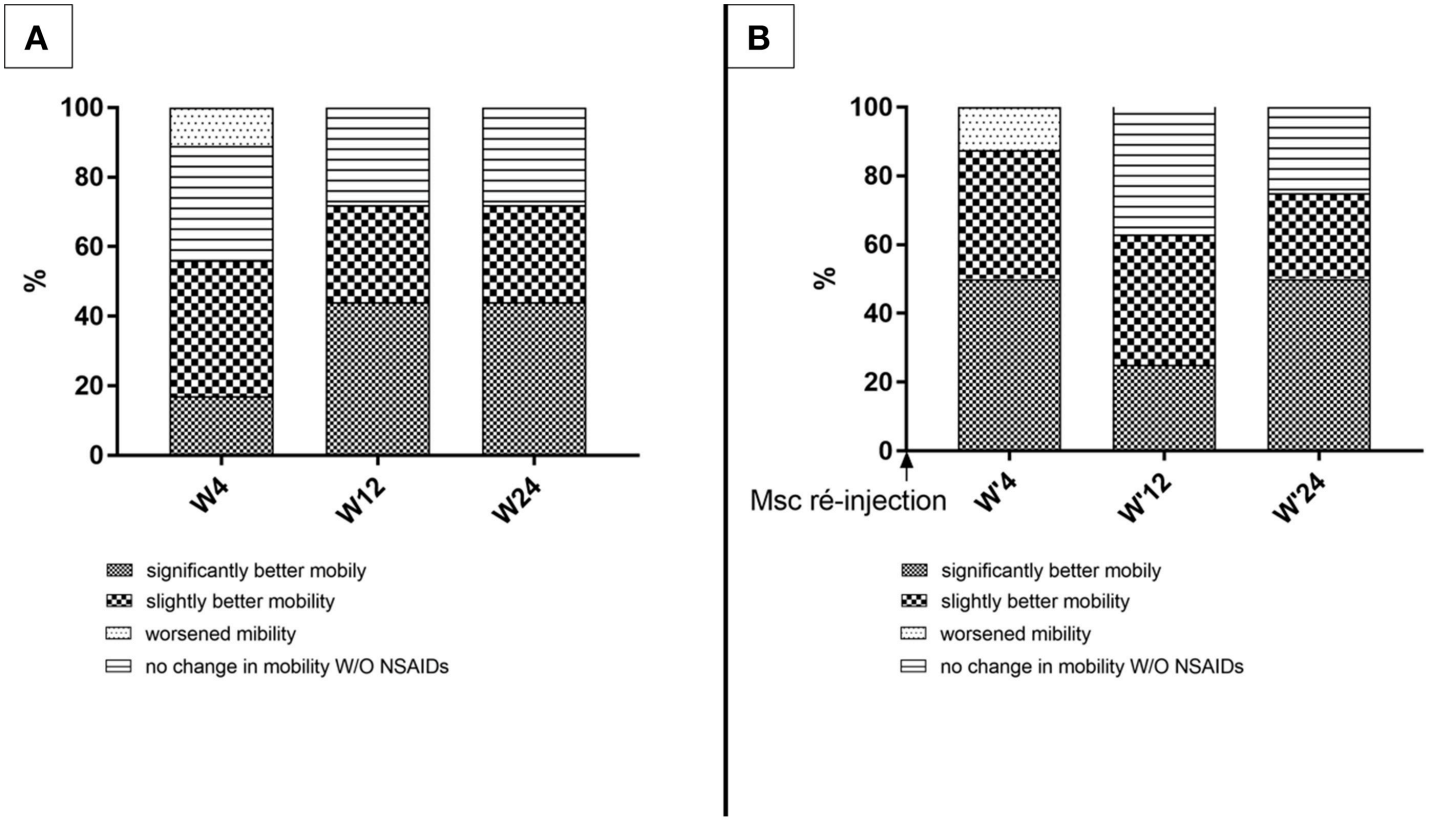
Owner's evaluation of dog's mobility. These figures show the owner's satisfaction **(A)** following the first injection (*n* = 16 dogs); **(B)** following the second injection (*n* = 8 dogs).

During the course of the study, owners were asked to evaluate their dog's mobility at each of the follow up time points (Figure 5A). Owner-assessments showed improvements during the course of the study. At 4 weeks post-injection, the dog's mobility was reported as improved by more than 50% of the owners [clear improvement (17%), mild improvement (39%)], while two owners reported transiently worsened mobility of their animals. At 3 and 6 months, 75% of owners reported mobility benefits [significantly improved (44%), slightly improved (28%)].

Dogs re-injected at M6 (*n* = 8) were evaluated once again by their owners according to the same 4-grade scale (Figure 5B). Only one owner reported worsened mobility of their dogs at W'4. The proportion of owners reporting improvement of their animal's mobility reached 88% [clear improvement (50%), mild improvement (38%)] 4 weeks post-treatment. This percentage was 63% at W'12 and 75% at W'24.

### Coherence Appraisal Between Veterinary Practitioner and Pet Owner Evaluations

During the first follow up visits (W4, W12, W24), only 3/16 evaluations were divergent at W4, 1/16 at W12, and 0/16 at W24. For dogs re-injected (*n* = 8), 1 over 8 evaluations was divergent at all-time points of the follow up (W'4, 12', 24'), and 1 evaluation was divergent only at W'24.

Two years post-injection, the rate of owner's satisfaction reached 75% (7 owners very satisfied; 5 owners satisfied). One owner was unsatisfied, while 3 owners declined to answer the survey. Owners were also asked to evaluate any lameness relapses of their pet during the 2 years following injection. Six owners reported occasional lameness after sustained physical activity. The requirement for NSAID during this period was recorded. Among the 6 dogs who were taking NSAID before MSC injection only 2, both with elbow OA (#10, #15) had to resume treatment during the 2 years period but only on a need-given basis and 4 (#1, #3, #5, #13) remained NSAID free. Two dogs (#7, 16) which weren't on NSAID had to initiate NSAID treatment during the 2 years period, however not during the 6-month veterinarian clinical follow up period. Five were still NSAID free 2 years after MSC treatment.

### Immunological Analyses

A positive control, consisting in a polyclonal serum generated by immunization of rabbits with canine MSC lysate from the different batches used in this study (cf. Materials and Methods), was tested to validate the analytical method. A strong difference of fluorescence intensity was detected between the fluorescence intensity signals of the immunized serum and the pre-immune serum (Table 3 and Supplementary Figure 2A). This confirmed the sensitivity and specificity of the method to detect canine alloantibodies.

**Table 3:** Flow cytometry antibody binding results.

**MFI**		**Basal MSC**				**IFN-Primed MSC**			
	**DOG ID**	**Cut-off of positivity**	**Δ(W4-D0)**	**Δ(W12-D0)**	**Δ(W24-D0)**	**Cut-off of positivity**	**Δ(W4-D0)**	**Δ(W12-D0)**	**Δ(W24-D0)**
1st Injection	#8	46,149	ND	10,552	−26,924	159,689	ND	−16,222	−12682
	#11		699	4,577	ND		50,762	−88,265	ND
	**#5**		26,443	12,989	17,208		−33,122	−75,189	−56,500
	#3		ND	−15,529	377		ND	22,078	−11,055
	**#4**		15,483	−7,058	ND		104,221	−8,551	ND
2nd Injection	#12	100,709	3,976	1,555	−12,531	320665	58,822	−1878	37,845
	#14		ND	−27,533	−58,628		ND	69,975	−45,118
	#7		ND	−3,459	−8,014		ND	−144,775	3,311
	**#4**		96,843	−33,059	−388		84,059	**562,119**	244,919
	**#5**		735	−1,168	−690		ND	ND	ND
Positive control (rabbit)		25,746	Δ(D42–D0)						
	**Cell line 1**		**1,987,286**						
	**Cell line 2**		**4,307,566**						

Following this validation step, 10 dogs having pre- (D0) and post-MSC (W4, W12, W24) administration serum samples were used for cross-match assay by flow cytometry. Among these, 5 dogs were treated with a single injection of cells, and 5 dogs beneficiated from a repeated injection of MSC 6 months apart. For 2 dogs (#4 and #5), sera collected after the first and second injections were available.

No antibody response could be detected against MSC following the first injection even when exacerbating MSC immunogenicity by priming donor MSC with IFN-γ prior to incubation with recipient serum (Table 3 and Supplementary Figure 2B). Only 1 dog (dog#4) displayed a significant positive signal in cross-match assay at W12 following the second injection and only with IFN-γ-primed MSC (Table 3 and Supplementary Figure 2C).

## Discussion

### Study Design

This pilot study was conducted as a compassionate (absence of any satisfactory therapeutic options), monocentric open and uncontrolled study. Considering the severity of OA in the dogs' cohort and the failure of standard of care treatments, ethical committee did not allow to include a placebo control group in this pilot study.

Since MSC have an anti-inflammatory effect, any co-treatment with NSAID, even if not efficient, could have a confounding effect. Recent clinical trials on large cohorts of OA dogs unfortunately do not mention the presence of such concurrent therapies before or during the study, blurring meaningful conclusions (8, 9). Results from our previous study (12) comparing MSC and anti Cox2 treatment effects on lameness and pain after joint surgery justified that this study could be performed without any concurrent anti-inflammatory treatment. However, if clinical conditions significantly worsened during the study, opioid receptor agonists were permitted. If NSAID was prescribed during the 6-month follow up, it was considered as an exclusion criterion from veterinary clinical evaluation.

Studies employing allogeneic MSC in OA generally use one batch of cells from a single donor (8). In our study, we intentionally used different batches of MSC to integrate inherent batch to batch cell variability bias. In most of the animal cell therapy studies, cellular characterization relies on viability, phenotype and differentiation potential. There are few predictive assays relevant for the immunomodulatory effect of MSC. Only Harman and coll. report the use of an undisclosed predictive assay. In our study, we used IDO enzymatic activity assay as recommended by the last ISCT consensus (24) as the release criteria of MSC batches. Furthermore, by using different batches we increased the possibility of detecting potential immune responses as no DLA typing could be performed to evaluated degree of tissue antigen mismatch between MSC and recipient.

For radiographic grading of OA elbows, EIWG was used as the recognized scoring system (29) established mainly for large dogs (i.e., Berneses, Labradors, Retrievers etc.,). In our study most of dogs with elbow OA were small-medium size. Osteophyte size in EIWG scale were deemed poorly applicable to those dogs. Therefore, the same radiographic scoring system was used for both hip than elbow (30, 31). The higher grade of severity was recorded if front and profile incidence gave different scores.

Both clinical and radiographic findings were integrated to grade OA severity, as recommended by the recent recommended OA clinical scoring system (21). Some studies only rely on radiographic findings to diagnose OA (9); while others only clinical findings (8). However, radiographic findings sometimes do not correlate well with clinical symptoms (32). Therefore, dogs were eligible if joints were graded with moderate or severe OA either clinically or radiographically.

### Clinical Safety

#### 1—Short Term Safety (48 h)

In our study, where dogs had no NSAID support, 5/22 joints displayed mild to moderate local joint discomfort and pain immediately 24 h after the first injection. For dogs receiving second injection in the same joint, 6/11 joints (mainly hips) displayed this clinical sign. In each situation, clinical signs disappeared within 48 h after injection. In their study Harman and coll. did not report any AE, except for one dog with joint pain in the control group (8). However, their study design may underestimate incidences since NSAID treatments were not discontinued upon inclusion. Shah and coll. may have missed those short-term AEs since in their study dogs were discharged only 2 h after injection. These findings corroborates our previous results (12) showing similar adverse event (AEs) shortly after injection. Nonetheless, this short-term AE might be more related to the injection procedure itself rather than to cell product, since, 1—both in this study and the one performed by Taroni et al. (12) MSC were administered without NSAID. 2—AEs were more frequently reported for hips which are more difficult to inject than other joints, 3—AEs occurred in only one joint in dogs receiving bilateral injections with the same MSC product. Such AEs may prompt practitioners to prescribe a short course of morphinomimetics for 3–5 days after MSC injection.

#### 2—Medium-Term Safety (48 h-6 Months)

No AEs was evidenced throughout the 6 months period.

#### 3–Long-Term Safety (6 Months−2 Years)

Human long-term safety studies have been well-documented (14) but are lacking in animal trials. Taking into account the underestimation bias introduced by owners not answering safety assessments, getting a high response rate, is critical. In our study 95% owners answered on average up to 2 years AEs safety surveys making our assessment more reliable. Records for long-term safety (2-years), after one or two injections, did not indicate other MSC-related AEs, such as infectious disease or neoplastic processes that could be attributed to unspecific immunomodulatory effects of MSC treatment or chromosomal abnormalities acquired during *ex-vivo* cell expansion processes (33). Two boxers aged 9 and 8 years old had suspicion of a testicular tumor and mastocytoma, respectively (dog#1 and dog#3). Although confirmatory histology would have been informative, testicular cancer is the second-most common form in older pure bred dogs and particularly in Boxers (34–36). Furthermore, although it is not possible to definitely exclude the hypothesis of an at-distance systemic immunomodulatory effect of MSC on existing tumoral processes or the migration of abnormal MSC out of the joint to ectopic tissues, the hypothesis is weak since MSC injected intra-articularly have not been detected in general circulation and immunomodulatory effect is believed to be transient for allogeneic MSC. In a retrospective safety study with OA dogs having received total hip replacement, 30% of dogs developed tumors during within 4 years and neoplasia was the main cause of death (37). One case of desmodecis was reported. The restricted population in our study is not amenable to accurately evaluating a risk with intrinsic low incidence.

### Time Course Evaluation of Recipient Immunological Response Against MSCs

In our work, no alloantibodies could be detected at any time point in recipients' serum against single or repeated administration of native neonatal allogeneic MSC, either by IgG or IgM analysis (data not shown) or cross-match. However, it is hardly conceivable that, despite MSC immune-evasiveness and joint relative insularity, an immunocompetent recipient may not elicit an immune response at all against “non-self” MSC especially in an inflammatory joint where synovial membrane is particularly vascularized. Therefore, we tested again dog recipient sera against the same MSC batches, but after the latter being purposely primed *ex vivo* with recombinant IFN-γ cytokine to increase their immunogenicity. We confirmed that IFNγ pre-treatment effectively increased MHC-I and II molecule expression at MSC surface. Interestingly, levels of MHC-II induction appear to be modest compared to those obtained by others with bone marrow-derived MSC (38). This could be related to a lower intrinsic immunogenicity of neonatal MSC, as described by Prasanna et al. (39) In this specific context, only dog#4 showed a mild positive signal with cross-match assay at W12 after second injection. No information could be retrieved from dog#4 electronic records that could be considered as a confounding factor in the interpretation of this positive signal (i.e., infection, vaccine, pregnancy…) leaving the hypothesis of a humoral response against allogeneic MSC valid. The absence of detection of positive signal in cross-match assay for other dogs could be attributed to a better DLA matching for these dogs, although this is unlikely. Unfortunately, the absence of DLA typing data prohibits further interpretation. It would have been interesting to explore the biological functionality of those antibodies in dog#4 such as complement-dependent cytotoxicity as performed by other groups (19, 20). The relative low number of dogs having received a second injection and for which we had serum samples, preclude to draw definitive conclusion about detrimental or beneficial clinical consequence of these findings. Furthermore, since dog#4 had a good clinical evolution, free of NSAID, and without lameness recurrence over the 24-month period, there may be no direct correlation between the detection of a humoral response and a deleterious effect on clinical status as suggested by Pezzanite et al. (19). In a recent human clinical study evaluating allogeneic MSC in knee OA, although the authors were able to detect specific antibodies specific to MSC in 2/13 patients their clinical evolution was, as in our case, not worsened compared to MSC with higher degree of HLA matching (40). A recent study by Dazzi F. group in human GvHD demonstrated elegantly that allogeneic MSC needed to undergo apoptosis triggered by recipient's lymphocyte response against MSC, to deliver immune modulation and clinical efficacy (41). Taken altogether, those results justify evaluating DLA matching degree and humoral and cellular immune response to better enlighten clinical evolution and maybe mechanism of action.

### Efficacy

Taken together there was significant clinical improvement assessed by practitioners at all-time points after MSC treatment. A majority of owners attested to marked improvement as in other published studies (8, 9). Interestingly, practitioner and owner evaluations were coherent at the mid- and end-point of the follow up (W12 and W24), strengthening analysis of our selected outcome variables.

Despite the limited number of joints, sub analysis by joint (hip vs. elbow) is informative. In both cases, significant clinical improvement was shown at W12, while it was significant at W24 only for hips. Interestingly, after a second injection, clinical efficacy was restored up to W'24 in any joints.

Our study did not include an objective measurement such as dynamic gait evaluation since different types of appendicular joints were evaluated and some dogs had bilateral OA, rendering data not comparable. Vilar and coll. used plate force to measure MSC efficacy at 1 month only; but all dogs were treated for coxofemoral arthrosis and only data from the more lame limb was analyzed (42). Objective measurement would require a control placebo group as a fixed reference to be able to compare with lame dogs treated with MSC (supposed to improve) or with placebo (supposed to worsen). Another limit of the proper interpretation of the efficacy data is the lack of blinding of owners and referring vets that may have result in biased clinical and owners' evaluation.

In our study, the dog's age may influence the extent of clinical improvement as noted by Shah et al. (9) but group size limited our conclusions (data not shown). However, in accordance with other studies, joints associated with higher scores of lameness have higher positive response rates than less lame ones (28, 42). Furthermore, as previously reported (42), dogs with unilateral OA displayed more evident clinical scoring improvement over 3–6 months than dogs with bilateral OA (data not shown).

86% owners answered questions related to lameness (i.e., recurrence/persistence) and/or detailed pharmacological OA treatment taken during the period. Results suggest that while persistent lameness was decreased by MSC in dogs with later stage OA, requirement for NSAID support can be reduced or even suppressed (*n* = 2). MSC are an attractive alternative in situations when NSAID are not an option and can provide owner satisfaction (12/13).

### Significance to Clinical Practice

This study is the first one reporting the use of neonatal derived MSC in client owned dogs with moderate to severe OA. Healthy neonatal tissues are an attractive source of MSC compared to adult tissue. Their procurement doesn't raise any ethical concerns (43, 44). The risk of transferable infectious diseases is low (45) (EMA/CVMP/ADVENT/803494/2016). In addition, age-related product variability is much less compared to autogenic MSC (46). Besides which, the amount of MSC that can be obtained from those tissues makes them amenable for industrial scale up.

This prospective pilot study is the first one to suggest the long-term safety and efficacy of single or repeated IA administration of canine neonatal MSC in dogs with moderate to severe OA. When considering field clinical studies with client-owned animals, long-term clinical study is a challenge for the investigational team. In fact, several situates may aggregate to break the link with the owner, such as owners moving to another region or a change of the dog's vet, or just the unwillingness of the owner to accept the cumbersome constraints of a clinical protocol. However, we have demonstrated the feasibility of implementing a successful long-term active follow-up study with a low rate of “lost from follow-up.”

## Conclusion

This study provides additional evidence for the long-lasting effect of MSC therapy in alleviating pain and lameness in dogs with moderate or severe OA and stabilization of the disease progression over a 2-year period. Both subjective assessments (veterinarian examinations and owner's evaluations) were coherent to suggest efficacy of the therapy for OA management. The absence of alloantibodies following one or two IA injections of neonatal allogeneic MSC suggests that this therapeutic approach is well-tolerated and can be repeated if required. These promising results allow veterinarians to consider MSC as an alternative to NSAID in the management of late-stages of OA when NSAID are inadequate or contraindicated.

## Author Contributions

The authors on this paper qualify as having provided the following overall contributions: EV, QC, TC, CC, PP, VL, all acted as study site investigators, by recruiting patients, performing treatment, and clinical evaluation. EV overviewed all the clinical and surgical aspects of the study, interpreted imaging data and participated in review of the manuscript. PS gave critical advices on cross-matches techniques and allogeneic immune responses, as well as having participated in analysis of cross-match results. NS performed end process controls on cells batches, was responsible for cross-match techniques and contributed to manuscript writing. CR was in charge of producing cell batches and in process controls. MF directed the study, coordinated follow up visits and worked on statistical analysis and manuscript writing. NP contributed to manuscript submission and statistical analysis. NG participated in analysis of the results and manuscript writing. SM provided cell products, participated in study design, results analysis, and manuscript writing. RR performed immunological assays.

### Conflict of Interest Statement

SM is a current and principal shareholder of Vetbiobank. MF, NS, CR, NP, and RR are current employees of Vetbiobank. The remaining authors declare that the research was conducted in the absence of any commercial or financial relationships that could be construed as a potential conflict of interest.

## Supplementary Material

Supplementary Data 1 Survey filled up by the owners at 2 years.

Supplementary Table 1 Parameters used to grade joint OA and scale (0–4) of evaluation.

Supplementary Table 2 Details of the dogs re-engaged for 6months follow-up after a second injection in a same member.

Supplementary Figure 1 Effect of IFN-γ on Major Histocompatibility Complex (MHC) molecule and indoleamine 2,3-dioxygenase (IDO) expression in canine MSC. **(A)** Gating strategy of the MSC population. **(B)** MHC-I (left) and MHC-II (right) expression of IFN-γ primed MSC (red: control isotype, blue: unstimulated MSC, orange: IFN-γ primed MSC). **(C)** MHC-I expression of IFN-γ primed MSC using canine serum blocking conditions (red: isotype control, blue: unstimulated MSC, orange: IFN-γ primed MSC). **(D)** IDO activity of untreated (blue) and IFN-γ primed (orange) MSC was evaluated using Ehrlich test and analyzed by 450 nm spectrophotometer.

Supplementary Figure 2 Representative figures of the crossmatch analysis. **(A)** Crossmatch procedure validation with a polyclonal serum obtained from rabbits immunized with canine MSC compared to the pre-immune serum. Mean fluorescence intensity (MFI) is given in each dot plot. The histogram (right hand side panel) allows to compare the fluorescence intensity obtained with the pre-immune serum (red curve) and with the positive control (blue curve). **(B)** Representative flow cytometric analysis of serum collected from dog#4 [D0 and week 12 (W12) time points] following the first injection of MSC. MFI is given in each dot plot. Histogram overlays (right hand side panel) do not show a shift of the fluorescence intensity. **(C)** Flow cytometric analysis representative of the dog #4 following the second injection of MSC (D0 and W12 time points). Histogram overlays show a shift of the fluorescence signals, corresponding to the detection of alloantibodies. [red: day 0 (D0); blue: week 12 (W12)].
