# Supplementary material for: Long-Term Safety and Efficacy of Single or Repeated Intra-Articular Injection of Allogeneic Neonatal Mesenchymal Stromal Cells for Managing Pain and Lameness in Moderate to Severe Canine Osteoarthritis Without Anti-inflammatory Pharmacological Support: Pilot Clinical Study
Source: Front Vet Sci. 2019 Feb 5;6:10. doi: 10.3389/fvets.2019.00010 (PMC6371748; doi:10.3389/fvets.2019.00010)
Supplement: Supplementary Data 1 — Survey filled up by the owners at 2 years. [file Data_Sheet_1.doc]

OWNER QUESTIONAIRE

A two-year canine clinical follow up study

| OWNER : |
| --- |
| Surname : ……………………………………………....  Forename : …………………………………………..  Telephone No. : …………..............................  E-mail : ................................................  Name and address of prescribing veterinarian :  …………………………………………………………............................................................................................................................................................................................................................................................................................ |
| ANIMAL : |
| Name : ………………………...........  Breed : …………………………………  Sex : ................................... |
| THE ANIMAL’S CONDITION : |
| Weight: ……………………….  General state of health: …………………………………  Other current treatments: □ No □ Yes, please detail: ............………………………………………………………………. |
| INJECTION OF MESENCHYMAL STEM CELLS: |
| Reason for treatment: ………………………………………………………………………………………………  Number of injections: □ 1 □ 2 □ 3 Other: …………..  Date(s) of the injections: …………………………… |
| UNDESIRABLE SIDE EFFECTS: |
| Have you noticed anything abnormal about your animal that has developed between 6 months after  treatment and the present day?  □ No  □ Yes  If yes, please describe the symptoms, when you first noticed them, the time they persisted for and did you take your animal to the vet?  .....................................................................................................................................................................................................................................................................................................................................................................................................................................................................................................................................................................................................................................................................................................................................................................................................................................................................................................................................................................................................................................................................................................................................................................................................................................................................................................................................................................................................................................................................................................................................................................................................................................................................................................................................................................................................................................................................................................................................................................................................................................................................................................................................................................................................................................................................................................................................................................................................................................................................................................................................................................................................................................................................................................................................................................................................................................................................................................................................................................................................................................................  How have these side effects evolved?  □ Favourably □ Unfavourably □ Deceased  Have you noticed the re-appearance of the same symptoms responsible for treatment by stem cell?  □ No □ Yes  If yes, about how long after the treatment did they arise? .............................................................................  Please describe the intensity of the symptoms:  □ Better than before the treatment □ About the same □ Worse than before |
| TREATMENT EFFICACY : |
| On a scale of 1 to 10 how would you rate the efficacy of the treatment?  □ 0 □ 1 □ 2 □ 3 □ 4 □ 5 □ 6 □ 7 □ 8 □ 9 □ 10  Similarly, how strongly would you recommend the treatment?  □ 0 □ 1 □ 2 □ 3 □ 4 □ 5 □ 6 □ 7 □ 8 □ 9 □ 10 |
| ANY ADDITIONAL COMMENTS :  ....................................................................................................................................................................................................................................................................................................................................................................................................................................................................................................................................................................................................................................................................................................................................................................................................................................................................................................................................................................... |
