# Supplementary material for: Long-Term Safety and Efficacy of Single or Repeated Intra-Articular Injection of Allogeneic Neonatal Mesenchymal Stromal Cells for Managing Pain and Lameness in Moderate to Severe Canine Osteoarthritis Without Anti-inflammatory Pharmacological Support: Pilot Clinical Study
Source: Front Vet Sci. 2019 Feb 5;6:10. doi: 10.3389/fvets.2019.00010 (PMC6371748; doi:10.3389/fvets.2019.00010)
Supplement: Supplementary Table 1 — Parameters used to grade joint OA and scale (0–4) of evaluation. [file Table_1.docx]

| Parameter | Notation |  |
| --- | --- | --- |
| Lameness | 1 | Walks normally No lameness |
|  | 2 | Intermittent weight-bearing lameness |
|  | 3 | Permanent weight-bearing lameness |
|  | 4 | Non weight-bearing lameness |
| Pain (palpation-pression) | 1 | No pain |
|  | 2 | Mild signs, dog turns head in recognition |
|  | 3 | Moderate signs, dog pulls limbs away |
|  | 4 | Severe signs dog vocalizes or bites |
| Pain (Mobilization) | 1 | No pain |
|  | 2 | Mild signs, dog turns head in recognition or pull slightly the leg |
|  | 3 | Moderate signs, dog pulls firmly limbs away |
|  | 4 | Severe dog vocalizes or bites |
| Heat | 1 | No warmer than contralateral limb |
|  | 2 | Mildly warmer |
|  | 3 | Moderately warmer |
|  | 4 | Markedly warmer |
| Total score | /16 | Lameness + Pain (palpation-pression + mobilization) + Heat |
