# Supplementary material for: Long-Term Safety and Efficacy of Single or Repeated Intra-Articular Injection of Allogeneic Neonatal Mesenchymal Stromal Cells for Managing Pain and Lameness in Moderate to Severe Canine Osteoarthritis Without Anti-inflammatory Pharmacological Support: Pilot Clinical Study
Source: Front Vet Sci. 2019 Feb 5;6:10. doi: 10.3389/fvets.2019.00010 (PMC6371748; doi:10.3389/fvets.2019.00010)
Supplement: Supplementary Table 2 — Details of the dogs re-engaged for 6months follow-up after a second injection in a same member. [file Table_2.docx]

| Dog ID | 1st injection | 2nd injection |
| --- | --- | --- |
| #4 | 2 elbows | 2 elbows |
| #5 | 1 elbow | 1 elbow |
| #6 | 1 hip | 1 hip |
| #7 | 2 hips | 2 hips |
| #8 | 1 hip 1 carpal joint | 1 hip |
| #10 | 2 elbows | 2 elbows |
| #12 | 1 hip | 1 hip |
| #14 | 1 hip | 1 hip |
