# Supplementary figures and images for: Long-Term Safety and Efficacy of Single or Repeated Intra-Articular Injection of Allogeneic Neonatal Mesenchymal Stromal Cells for Managing Pain and Lameness in Moderate to Severe Canine Osteoarthritis Without Anti-inflammatory Pharmacological Support: Pilot Clinical Study
Source: Front Vet Sci. 2019 Feb 5;6:10. doi: 10.3389/fvets.2019.00010 (PMC6371748; doi:10.3389/fvets.2019.00010)

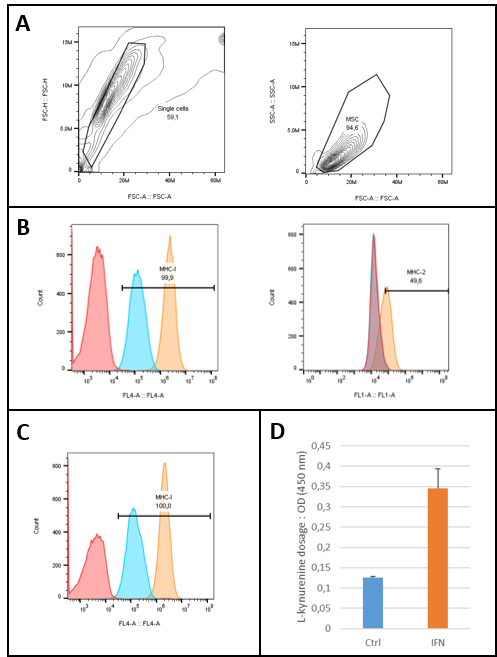

Supplement: Supplementary Figure 1 — Effect of IFN-γ on Major Histocompatibility Complex (MHC) molecule and indoleamine 2,3-dioxygenase (IDO) expression in canine MSC. (A) Gating strategy of the MSC population. (B) MHC-I (left) and MHC-II (right) expression of IFN-γ primed MSC (red: control isotype, blue: unstimulated MSC, orange: IFN-γ primed MSC). (C) MHC-I expression of IFN-γ primed MSC using canine serum blocking conditions (red: isotype control, blue: unstimulated MSC, orange: IFN-γ primed MSC). (D) IDO activity of untreated (blue) and IFN-γ primed (orange) MSC was evaluated using Ehrlich test and analyzed by 450 nm spectrophotometer. [file Image_1.TIF]

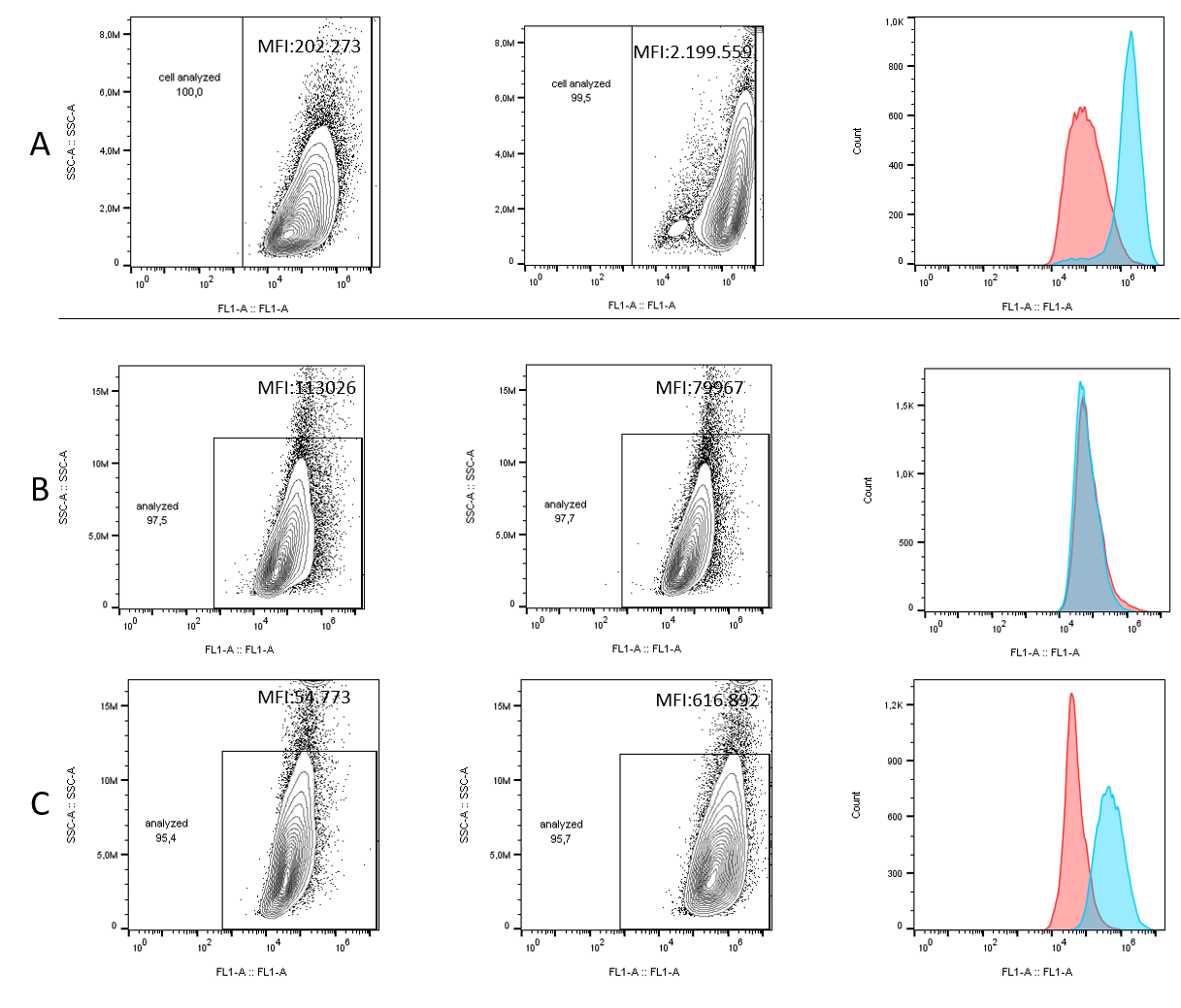

Supplement: Supplementary Figure 2 — Representative figures of the crossmatch analysis. (A) Crossmatch procedure validation with a polyclonal serum obtained from rabbits immunized with canine MSC compared to the pre-immune serum. Mean fluorescence intensity (MFI) is given in each dot plot. The histogram (right hand side panel) allows to compare the fluorescence intensity obtained with the pre-immune serum (red curve) and with the positive control (blue curve). (B) Representative flow cytometric analysis of serum collected from dog#4 [D0 and week 12 (W12) time points] following the first injection of MSC. MFI is given in each dot plot. Histogram overlays (right hand side panel) do not show a shift of the fluorescence intensity. (C) Flow cytometric analysis representative of the dog #4 following the second injection of MSC (D0 and W12 time points). Histogram overlays show a shift of the fluorescence signals, corresponding to the detection of alloantibodies. [red: day 0 (D0); blue: week 12 (W12)]. [file Image_2.TIF]
